# 

**Published:** 1869-07

**Authors:** 


### Books and Pamphlets Received.

A Treatise on the Diseases of the Eye. By J. Soelberg, Professor of Ophthalmology in King's College Hospital, London, Ophthalmic Surgeon to King's College Hospital, and Assistant Surgeon to the Royal London Ophthalmic Hospital, Moorfield. First American edition, with additions. Illustrated with 260 engravings on wood and six colored plates, together with selections from the test-types of Prof. E. Jaeger and Dr. H. Snellen. Philadelphia: Henry C. Lea, 1869. Received through and for sale by Theo. Butler & Son.

A Manual of Elementary Chemistry, Theoretical and Practical. By Geo. Fownes, F. R. S., late Professor of Practical Chemistry in the University College, London. From the tenth revised and corrected English edition. Edited by Robt. Bridges, M. D., Professor of Chemistry in the Philadelphia College of Pharmacy, with 197 illustrations. Philadelphia: Henry C. Lea, 1869. Received through and for sale by Breed & Lent, Buffalo.

A Practical Manual of the Treatment of Club-Foot. By Lewis A. Sayres, M. D., Professor of Orthopædic Surgery in Bellevue Hospital Medical College, etc., etc. New York: D. Appleton & Co., 1869. Received and for sale by Martin Taylor.

Prescription and Clinic Record. The Pocket-Book of every careful and progressive physician. New York: Wm. Wood & Co., 1869.

The American Exchange and Review. A Miscellany of Useful Knowledge and General Literature. Vol. 15, No. 3. Philadelphia: Fowler & Moon.

On Self-Enervation, its Consequences and Treatment. By C. S. Eldridge, M. D. (Homœopathic.) Chicago: C. S. Halsey, 1869.

Notes on the Phenols from Coal Tar, (the so-called Carbolic Acid,) and on Rhubarb. By E. S. Squibb, M. D.

Treatment of Lachrymal Affections. By Prof. Arlt, Professor of Ophthalmology at the University of Vienna. Philadelphia: Lindsay & Blakiston, 1869.

Henry C. Lea's Classified Catalogue of Medical and Surgical Publications, June, 1869.

Woman's Medical College of the New York Infirmary Annual Catalogue and Announcement, 1869.

Anniversary Oration delivered before the Medical Society of the District of Columbia, September 26, 1866. By J. M. Toner, M. D.

The Twenty-eighth Annual Announcement of the St. Louis Medical College, Winter Session, 1869–70.

Transactions of the Twentieth Annual Meeting of the Georgia Medical Association, April, 1869.

College of Physicians and Surgeons, New York. Medical Department of Columbia College. Sixty-second Annual Catalogue and Announcement, 1869.

The Proceedings of the Georgia Medical Association, Constitution and By-Laws.

